# Supplementary material for: Effect of Dietary Fructus mume and Scutellaria baicalensis Georgi on the Fecal Microbiota and Its Correlation with Apparent Nutrient Digestibility in Weaned Piglets
Source: Animals (Basel). 2022 Sep 14;12(18):2418. doi: 10.3390/ani12182418 (PMC9495044; doi:10.3390/ani12182418)
Supplement: Supplementary file 1 [file animals-12-02418-s001.zip › animals-1837679-supplementary.pdf]

Effect of dietary *Fructus mume* and *Scutellaria baicalensis* Georgi on the fecal microbiota and its correlation with apparent nutrient digestibility in weaned piglets

Feng Zhang[1, 2, *], Erhui Jin[1, 2, 3], Xiaodan Liu[1], Xu Ji[4], and Hong Hu[1]

[1]College of Animal Science, Anhui Science and Technology University, Chuzhou 233100, China; jineh@ahstu.edu.cn (E.J.); liuxd@ahstu.edu.cn (X.L.); huh@ahstu.edu.cn (H.H.)

[2]Anhui Province Key Laboratory of Animal Nutrition Regulation and Health, Chuzhou 233100, China;

[3]Anhui AnFengT Animal Medicine Industry Co., LTD, Hefei 230031, China;

[4]Anhui Province Key Laboratory of Livestock and Poultry Product Safety Engineering, Institute of Animal Science and Veterinary Medicine, Anhui Academy of Agricultural Sciences, Hefei 230031, China; jixu@aaas.org.cn

*Correspondence: zhangfeng@ahstu.edu.cn (F.Z.);

Journal name: Animals

Supplementary results for the fermented feed physiological characteristics

To explore the reasonable fermentation time and pH value of the fermented feed, we conducted fermentation treatments for the 14 groups of feeds in Table 1 and measured the pH values (Table S1). Among the 14 fermented groups, the pH was below 5.00 in the feeds fermented with the strains. Within these groups, as the fermentation time progressed, the pH of the 1% *Fructus mume* group fell to 3.83 and maintained at around 3.80 until the end of the fermentation; in the 3% *Fructus mume* group, the pH decreased from the beginning of fermentation and remained at 3.40 from 96 to 144 h; the pH of the 10% *Fructus mume*

group was around 3.40 during the fermentation. In the 3% *Scutellaria baicalensis* Georgi group, the pH trend was consistent with the 1% *Fructus mume* group. Within the groups fermented without the strains, the pH was always above 4.60 throughout the fermentation, which is not suitable for fermented feed.

Table S1. The pH values at different fermentation times (Mean ± SD).

	Fermentation time, h					
	24	48	72	96	120	144
Mixed substrates fermented with the fermentative strains						
Fructus mume						
1%	4.82 ± 0.02	4.37 ± 0.03	3.83 ± 0.02	3.89 ± 0.01	3.76 ± 0.03	3.81 ± 0.02
3%	3.72 ± 0.03	3.86 ± 0.02	3.55 ± 0.04	3.47 ± 0.02	3.39 ± 0.01	3.42 ± 0.02
10%	3.39 ± 0.01	3.42 ± 0.03	3.37 ± 0.02	3.35 ± 0.01	3.38 ± 0.03	3.40 ± 0.02
Scutellaria baicalensis Georgi						
1%	4.53 ± 0.03	4.56 ± 0.02	4.57 ± 0.02	4.53 ± 0.01	4.25 ± 0.03	4.12 ± 0.03
3%	4.50 ± 0.05	4.45 ± 0.03	3.93 ± 0.02	3.88 ± 0.03	3.90 ± 0.04	3.89 ± 0.02
10%	4.24 ± 0.03	4.11 ± 0.02	3.95 ± 0.01	3.92 ± 0.03	4.03 ± 0.02	4.16 ± 0.01
Control						
	4.47 ± 0.02	4.51 ± 0.01	4.41 ± 0.03	4.27 ± 0.02	4.19 ± 0.03	4.17 ± 0.01
Mixed substrates fermented without the fermentative strains						
Fructus mume						
1%	5.62 ± 0.02	5.22 ± 0.03	5.17 ± 0.01	4.88 ± 0.02	4.70 ± 0.03	4.67 ± 0.02
3%	5.54 ± 0.01	5.50 ± 0.04	5.48 ± 0.03	5.52 ± 0.03	5.51 ± 0.01	5.49 ± 0.02
10%	5.59 ± 0.01	5.53 ± 0.03	5.45 ± 0.03	5.39 ± 0.04	5.42 ± 0.02	5.40 ± 0.03
Scutellaria baicalensis Georgi						
1%	5.49 ± 0.03	5.52 ± 0.01	5.50 ± 0.02	5.54 ± 0.01	5.49 ± 0.02	5.50 ± 0.03
3%	5.51 ± 0.01	5.32 ± 0.03	5.21 ± 0.02	5.07 ± 0.02	4.89 ± 0.02	4.73 ± 0.03
10%	5.45 ± 0.03	5.46 ± 0.02	5.39 ± 0.03	5.43 ± 0.02	5.40 ± 0.03	5.41 ± 0.02
Control						
	5.57 ± 0.01	5.51 ± 0.03	5.33 ± 0.03	5.37 ± 0.02	5.41 ± 0.04	5.43 ± 0.01

Supplementary results for Krona analysis

The abundance statistics of each taxon were visualized using Krona, the results show the composition of microbial species at different taxonomic levels, the sector area corresponds to species abundance.

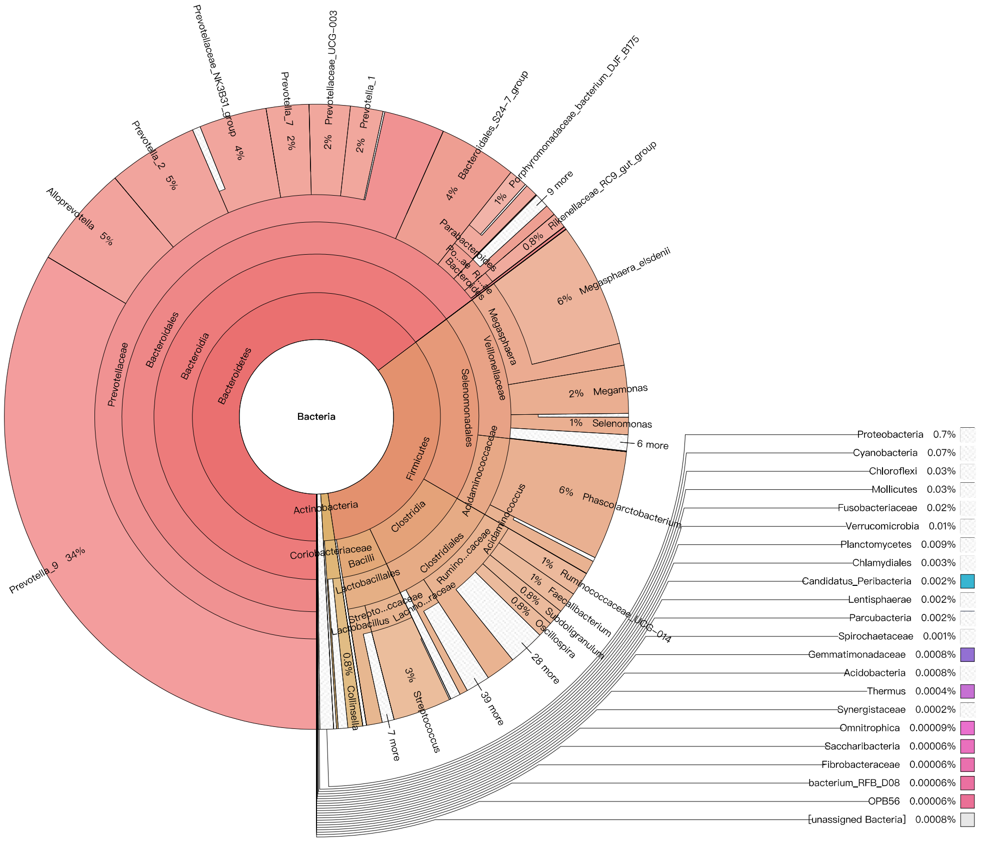

Figure S1. The total profiling of the composition of microbial species.

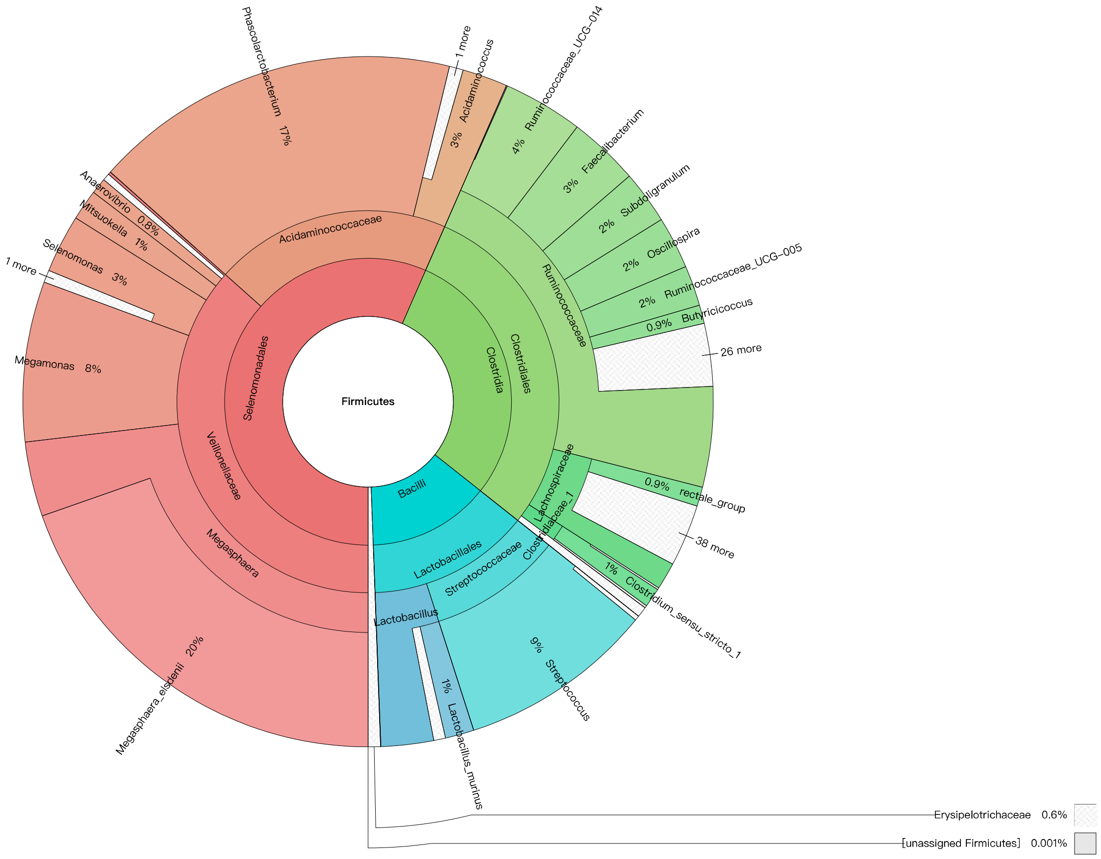

Figure S2. The composition of microbial species at the phylum level of *Firmicutes*.

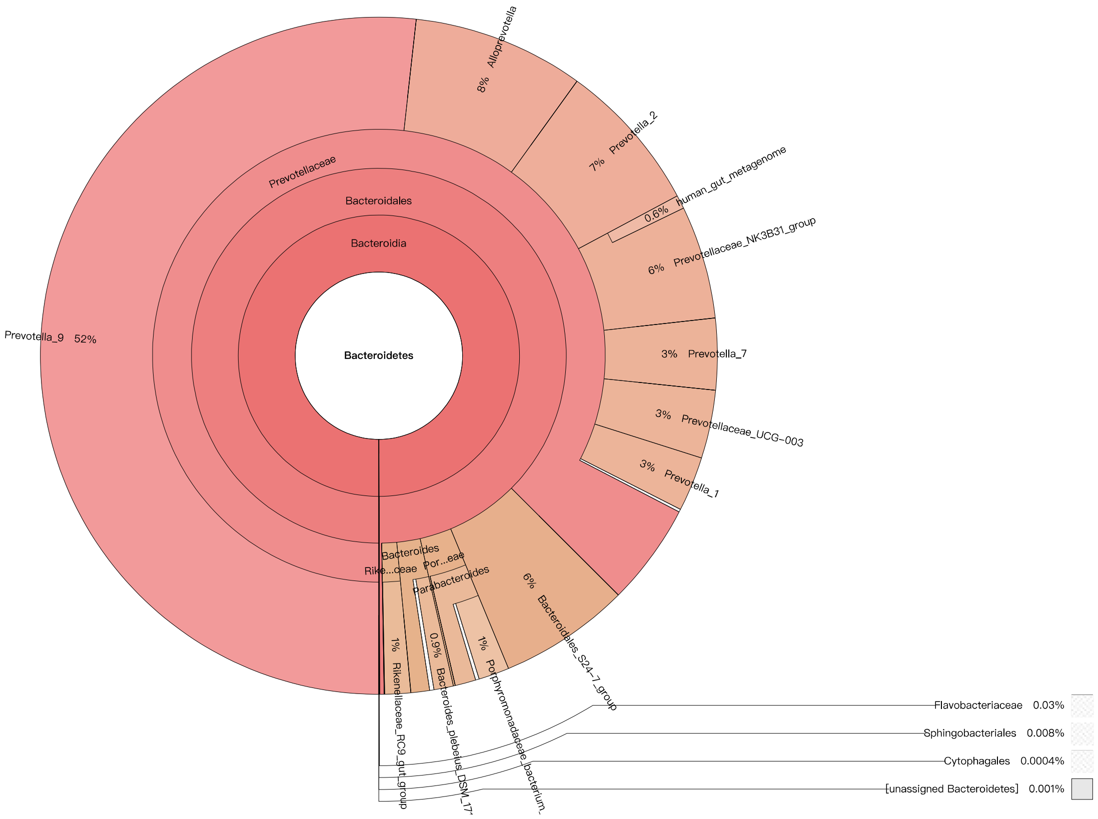

Figure S3. The composition of microbial species at the phylum level of *Bacteroidetes*.

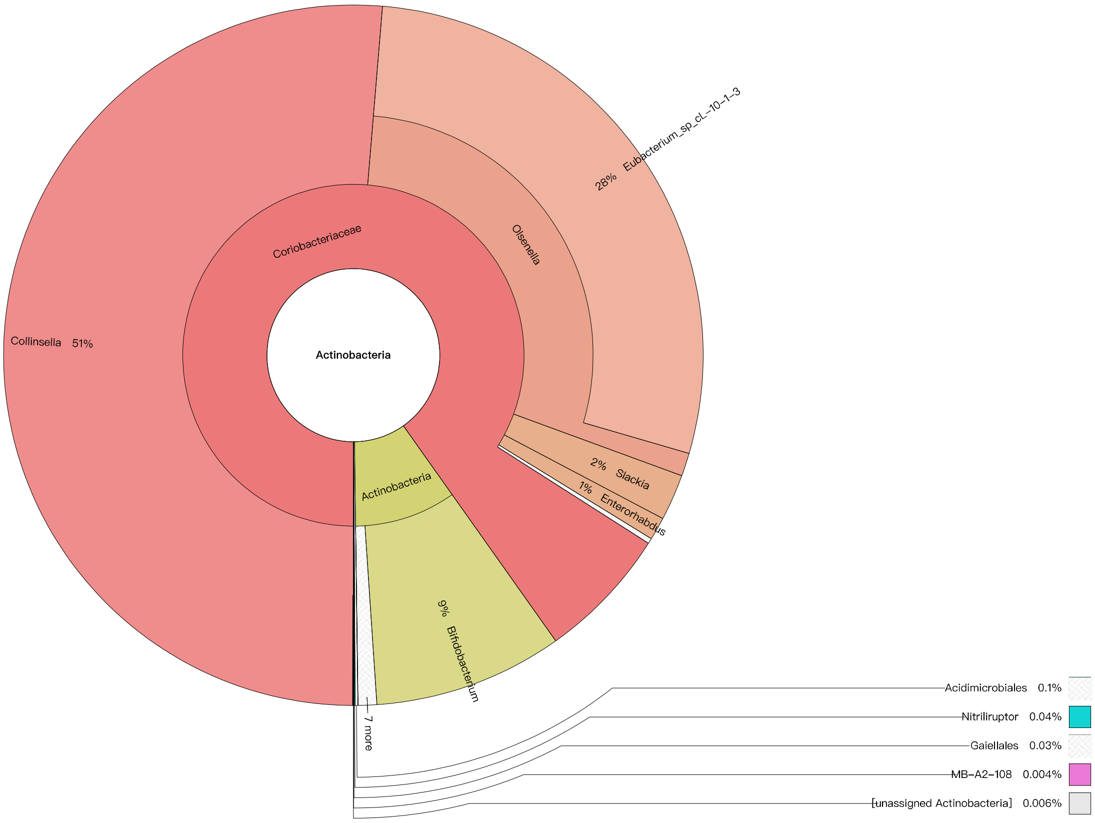

Figure S4. The composition of microbial species at the phylum level of *Actinobacteria*.
